# Measuring Physical Functioning Using Wearable Sensors in Parkinson Disease and Chronic Obstructive Pulmonary Disease (the Accuracy of Digital Assessment of Performance Trial Study): Protocol for a Prospective Observational Study

**DOI:** 10.2196/55452

**Published:** 2024-05-07

**Authors:** Debbie de Graaf, Nienke M de Vries, Tessa van de Zande, Janneke J P Schimmel, Sooyoon Shin, Nathan Kowahl, Poulami Barman, Ritu Kapur, William J Marks Jr, Alex van 't Hul, Bastiaan R Bloem

**Affiliations:** 1 Radboud University Medical Center Donders Institute for Brain, Cognition and Behavior Department of Neurology, Center of Expertise for Parkinson & Movement Disorders Nijmegen Netherlands; 2 Verily Life Sciences South San Fransisco, CA United States; 3 Radboud University Medical Center Radboud Institute for Health Sciences Department of Respiratory Diseases Nijmegen Netherlands

**Keywords:** Parkinson disease, COPD, chronic obstructive pulmonary disease, physical activity, physical capacity, wearable devices, walking, exercise, locomotion, home-based, wearable, wearables, wearable sensor, dementia, smartwatch, StepWatch, treatment

## Abstract

**Background:**

Physical capacity and physical activity are important aspects of physical functioning and quality of life in people with a chronic disease such as Parkinson disease (PD) or chronic obstructive pulmonary disease (COPD). Both physical capacity and physical activity are currently measured in the clinic using standardized questionnaires and tests, such as the 6-minute walk test (6MWT) and the Timed Up and Go test (TUG). However, relying only on in-clinic tests is suboptimal since they offer limited information on how a person functions in daily life and how functioning fluctuates throughout the day. Wearable sensor technology may offer a solution that enables us to better understand true physical functioning in daily life.

**Objective:**

We aim to study whether device-assisted versions of 6MWT and TUG, such that the tests can be performed independently at home using a smartwatch, is a valid and reliable way to measure the performance compared to a supervised, in-clinic test.

**Methods:**

This is a decentralized, prospective, observational study including 100 people with PD and 100 with COPD. The inclusion criteria are broad: age ≥18 years, able to walk independently, and no co-occurrence of PD and COPD. Participants are followed for 15 weeks with 4 in-clinic visits, once every 5 weeks. Outcomes include several walking tests, cognitive tests, and disease-specific questionnaires accompanied by data collection using wearable devices (the Verily Study Watch and Modus StepWatch). Additionally, during the last 10 weeks of this study, participants will follow an aerobic exercise training program aiming to increase physical capacity, creating the opportunity to study the responsiveness of the remote 6MWT.

**Results:**

In total, 89 people with PD and 65 people with COPD were included in this study. Data analysis will start in April 2024.

**Conclusions:**

The results of this study will provide information on the measurement properties of the device-assisted 6MWT and TUG in the clinic and at home. When reliable and valid, this can contribute to a better understanding of a person’s physical capacity in real life, which makes it possible to personalize treatment options.

**Trial Registration:**

ClinicalTrials.gov NCT05756075; https://clinicaltrials.gov/study/NCT05756075

**International Registered Report Identifier (IRRID):**

DERR1-10.2196/55452

## Introduction

Physical functioning is an important aspect of daily living. It represents a broad concept defined as one’s ability to carry out activities that require physical actions, ranging from activities of daily living to more complex activities that require a combination of skills within a social context. It is also known to be a powerful factor in the prevention and treatment of several health conditions in older adults [1-5]. Physical functioning is an independent predictor of functional independence, disability, morbidity, and mortality in healthy older people [4,5]. It is also relevant in a wide spectrum of people with chronic diseases [6-9], which makes it an important aspect to measure. Physical functioning requires multiple factors, including physical capacity and physical activity [1]. Physical capacity can be defined as what a person can do in a standardized environment, where physical activity is what a person truly does in daily life [10,11]. Today, physical capacity is measured in clinical settings using advanced tests like maximal oxygen uptake or walking tests such as the 6-minute walk test (6MWT) [1]. Validated questionnaires are generally used to obtain self-reported levels of physical activity in real-life situations [12,13].

Relying only on in-clinic standardized tests is suboptimal for several reasons. First, in-clinic tests give limited information on how a person is functioning in daily life. For example, people with Parkinson disease (PD) experience the so-called *kinesia paradoxa* in which they function unexpectedly well under stressful circumstances, which a clinic visit is for most [14]. Other symptoms such as tremors are typically worse during in-clinic assessments [15]. This problem is compounded by the fact that many people with PD take extra medication before their consultation hoping to make a good impression. Some even practice the clinical tests while sitting in the waiting room. Second, episodic visits are not well suited to detect the complex and evolving fluctuations in daily functioning [6,9,16]. Moreover, the brief in-clinic evaluations cannot reliably capture the common and disabling fluctuations in response to Parkinson medication [17]. Finally, measuring physical activity with a questionnaire is highly unreliable because of its retrospective and subjective nature [13].

With the advent of innovative technologies such as smartwatches, both aspects of physical functioning have become measurable in a person’s free-living environment [18-22]. Wearable technology may thus offer an attractive solution to overcome the aforementioned limitations of in-clinic testing [23]. This would be especially helpful for the evaluation of people with chronic conditions like PD or chronic obstructive pulmonary disease (COPD) because the symptoms of both diseases fluctuate within a single day per medication intake or environmental factors [17,24]. Deploying wearable sensors enables monitoring of daily physical functioning over a prolonged period, without relying on the person’s in-clinic performance or memory. Remote assessments of physical activity may also lower the barrier for people to check their performance regularly, and also lower the burden to do so for caregivers and researchers [25]. Wearable technology can also better evaluate the effectiveness of treatments, thus enabling a more personalized treatment. Today, measuring physical activity at home using wearable devices has become mainstream and is feasible [12,26,27]. More recently, also measuring physical capacity using wearables has been shown to be feasible but the clinimetric properties have been investigated to a very limited extent as yet [8,28-31].

Here, we propose a protocol to study a wrist-worn device that can measure physical activity and physical capacity passively and remotely while performing assessments at home. The measurement properties of this device regarding physical activity are good but have been obtained in a healthy population [32,33]. We will extend these results and focus on the measurement properties of measuring physical capacity using the 6MWT in people with PD and COPD.

Our primary aim is to study the measurement properties of a device-assisted 6MWT performed at home compared to in-clinic assessments. Our secondary aim is to study the clinimetric properties of the device-assisted Timed Up and Go test (TUG) at home compared to in-clinic assessments.

## Methods

### Study Design

**Figure 1:**
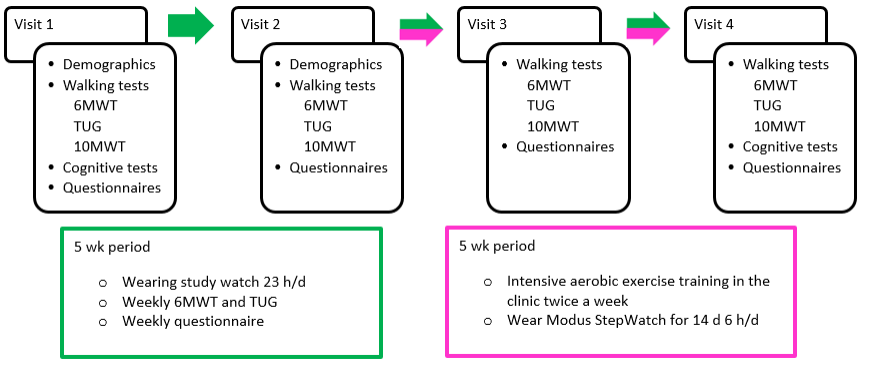
Study overview. 6MWT: 6-minute walk test; 10MWT: 10-meter walk test; TUG: Timed Up and Go test.

This is a decentralized, prospective, observational study with a 15-week follow-up involving participants with PD and COPD (Figure 1). This study will be coordinated by the Radboudumc and will be executed in 25 physiotherapy practices throughout the Netherlands. The physiotherapists will perform the in-clinic assessments. Participants will visit their local physiotherapy practice 4 times, once every 5 weeks, for in-clinic assessments of walking capacity, cognitive functioning, daily functioning, and specific disease symptoms. Data collection during each visit will take approximately 2 hours. During the in-clinic assessments, participants will wear 2 different wearable sensor devices; the Verily Study Watch on 1 wrist and the Modus StepWatch on the ankle. Participants will be instructed to wear the devices on the same side throughout this study. Moreover, during the course of this study, participants will wear the Verily Study Watch at home for 23 hours a day. Additionally, participants will perform 2 walk tests at home every week. Between visit 2 and visit 4, participants will participate in supervised, in-clinic, high-intensity exercise training according to the current physiotherapy guidelines [34,35]. During this training period, participants will wear the Modus StepWatch at home for 2 weeks. This will provide data to validate real-life walking measures from this study’s watch against the StepWatch. Throughout this study, a dedicated helpdesk will proactively assist participants, address problems and questions, and solve issues regarding this study and the devices. The Accuracy of Digital Assessment of Performance Trial (ADAPT) study is registered at ClinicalTrials.gov (NCT05756075).

### Study Population

We aim to enroll 200 participants, 100 with PD and 100 with COPD between December 2021 and July 2023. Participants are eligible for this study if they meet the following inclusion criteria:

PD or COPD diagnosed by a neurologist or pulmonologist, respectively; aged 18 years or older; able to read and understand Dutch; willing, competent, and able to comply with all aspects of the protocol, including follow-up schedule; willing and able to complete patient-reported outcomes via the internet; able to walk without the use of an assistive device; Hoehn and Yahr stage 1 or 2 (PD-specific); and COPD with perceived limited exercise capacity, irrespective of airway obstruction severity (COPD-specific).

Potential participants will be excluded if they have a high fall risk (for PD) or cardiovascular risk profile (for COPD) impacting the safety of performing the walk tests at home without supervision, as judged by the general practitioner. Patients with other comorbidities are explicitly not excluded from participation. Other exclusion criteria include a co-occurrence of PD and COPD, the inability to make an arm swing at all or if a patient is in a situation that prevents arm swing completely (eg, use of rollator), pregnancy, cognitive impairment as judged by the physiotherapist, participating in another investigational study, participating in a supervised high-intensity aerobic exercise program, or nickel allergy as components of the Verily Study Watch contain trace amounts of this metal.

### Recruitment and Enrollment

In each physiotherapy practice, 2 physiotherapists will participate, 1 specializing in COPD and 1 specializing in PD affiliated with the Dutch nationwide ParkinsonNet infrastructure [36]. The physiotherapists will be trained extensively and certified in performing all assessments of this study’s protocol to facilitate standardization between the participating practices. Up-to-date standard operating procedures will be shared with every practice as a reference and will be reviewed by the Radboudumc research team regularly. Further, the Radboudumc research team will guide and coach the physiotherapists to support adherence to this study’s protocol.

Physiotherapists will identify potentially eligible patients who are already known in their practice because of a former treatment episode or patients newly referred by the general practitioner or medical specialist and provide them with the information letter. If a patient shows interest in this study, a researcher from the Radboudumc will contact them providing additional information, address any questions, and check eligibility. When willing and eligible to participate, participants will be directed to Verily’s proprietary web-based system “Baseline Platform” where they are invited to create an account and digitally sign the screening consent form. Once the account is created, the physiotherapist will contact the participant’s treating physician to confirm the diagnosis and will ask about potential contraindications to participation in this study (ie, history of falling or cardiovascular risk). If the treating physician expresses no objections, the first appointment will be scheduled.

At the first visit, the physiotherapist will check eligibility again whereafter this study’s informed consent form will be signed on paper by both the participant and physiotherapist, who will perform the assessments. A copy will be provided to the participant. Participants who withdraw their participation during this study will be excluded from follow-up, but the data obtained will be kept in the central database and will be used for data analysis.

### Devices

#### Verily Study Watch

Throughout this study, participants with PD will wear the Verily Study Watch on the wrist of the least affected side while participants with COPD will wear the watch on the wrist of the nondominant side.

Participants will be asked to wear the Verily Study Watch preferably for 24 hours a day, except during daily charging and during wet activities (eg, showering or swimming). During the baseline visit, the physiotherapist will clarify the importance of the wear time with the participant and will explain and demonstrate the use of the Verily Study Watch. A paper-based manual plus an instructional video will be available for participants with instructions for the Verily Study Watch.

**Figure 2:**
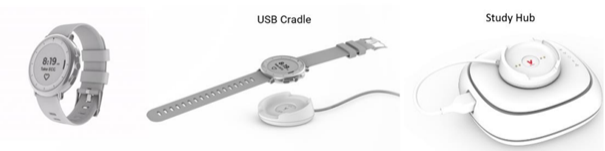
The Verily Study Watch, along with syncing and charging cradle and Verily Study Hub.

The Verily Study Watch is exclusively for investigational use in clinical trials only. It is a multi-sensor wearable device to extend data collection for clinical studies beyond trial sites and into the free-living environment. The watch contains an accelerometer, gyroscope, and a photoplethysmography. The device is not CE (Conformité Européenne) marked or cleared in the United States. The device enables the collection of physiological and environmental data about movement and activity, pulse rate, and skin impedance. Data from the Verily Study Watch will be encrypted and sent securely to the Verily Cloud using a USB synching and charging cradle and wireless connectivity bridge (Verily Study Hub; Figure 2). The Verily Study Watch has been deployed in several studies, including the Baseline Health Study [37], the Parkinson Progression Markers Initiative [38] in the United States, and the Personalized Parkinson Project [39] in the Netherlands.

#### Modus StepWatch

The Modus StepWatch will be placed on the ankle on the corresponding side of the Verily Study Watch during the 4 in-clinic visits. Between visit 2 and visit 4, participants will wear the Modus StepWatch at home for 14 consecutive days for at least 6 hours a day. Participants will be able to choose these 14 consecutive days themselves. To gain insight into the exact wearing times, the participants will create a log where they write down the exact times of wearing the Modus StepWatch.

This device is an ankle-worn wearable medical device (CE class 1 and marked medical in Europe) which will be used as a reference device for measuring participants’ ambulation. The onboard 2D accelerometer collects motion data. The collected data can be transferred to the corresponding StepWatch app installed on an iPad. From there, data will be transferred to this study’s database. The validity of this device has been obtained in people with PD [40] and COPD [41] as well as in healthy people [42-44].

### Assessments

#### Demographics

During the first visit, demographics, medical history, and medication intake will be registered. Cognitive functions will be assessed during the first and last visit. During each visit, several additional questionnaires on disease symptoms and quality of life will be completed (see Tables 1 and 2 for an overview).

**Table 1:** Overview of assessments performed by physiotherapist and questionnaires in the Accuracy of Digital Assessment of Performance Trial (ADAPT) study^a^.

Assessments			Visit 1		Visit 2^b^		Visit 3^b^		Visit 4^b^
**Motor functioning**									
	MDS-UPDRS-III/IV^c^ (PD^d^ only)	✓		✓		✓		✓	
	H&Y scale^e^ (PD only)	✓		✓		✓		✓	
	TUG^f^	✓		✓		✓		✓	
	6MWT^g^ (2×); pre- or post-Borg score fatigue and dyspnea	✓		✓		✓		✓	
	10MWT^h^ (Single context [3×], dual context [3×]	✓		✓		✓		✓	
**Cognitive functioning and quality of life**									
	MoCA^i^ (with alternative version during follow-up)	✓						✓	
	MDS-UPDRS-I (PD only)	✓		✓		✓		✓	
	EQ-5D-5L	✓		✓		✓		✓	
**Demographics and lifestyle**									
	Medical history	✓		✓					
	Medication	✓							
	Arm Hair Index	✓							
	Skin tone (Fitzpatrick Scale)	✓							
**Autonomic function**									
	Blood pressure (before 6MWT)	✓		✓		✓		✓	
	Heart rate (before 6MWT, 0 min after 6MWT, 1 min after 6MWT, 2 min after 6MWT)	✓		✓		✓		✓	
	SpO2^j^ (before 6MWT, 0 min after 6MWT, 1 min after 6MWT, 2 min after 6MWT)	✓		✓		✓		✓	

^a^Study Watch sensor data will be recorded during all in-clinic activities, including all walking tests. With the timestamps recorded on the Study Watch for the onset and offset of tasks via the Study Watch graphical user interface, a sensor-based 6-minute walk test will be estimated and compared to the site coordinator’s recorded actual 6-minute walk test.

^b^Every fifth week.

^c^MDS-UPDRS-I/II/III/IV: Movement Disorders Society–sponsored revision of the Unified Parkinson’s Disease Rating Scale (I) nonmotor aspects of experiences of daily living, (II) motor experiences of the daily living section, (III) motor examination section, and (IV) motor complications section.

^d^PD: Parkinson disease.

^e^H&Y scale: Hoehn and Yahr scale.

^f^TUG: Timed Up and Go test.

^g^6MWT: 6-minute walk test.

^h^10MWT: 10-meter walk test.

^i^MoCA: Montreal Cognitive Assessment.

^j^SpO2: oxygen saturation.

**Table 2:** Devices and at-home assessments.

Methods	Assessments	Time collected
Verily Study Watch	Photoplethysmography Heart rate IMU^a^ including a 3-axis accelerometer and 3-axis gyroscope Step count Skin impedance	Continuous data collection, up to 24 h/d
Modus StepWatch	Step count	All visits^b^
Self-reported patient questionnaires^c^	ESS^d^ MDS-UPDRS-II^e^ (Parkinson disease only) HADS^f^ PDQ-39^g^ (Parkinson disease only) LAPAQ^h^ CCQ^i^ (chronic obstructive pulmonary disease only)	After each visit
At home assessments (walking tests with Verily Study Watch)	Timed Up and Go test 6-minute walk test with pre- and post-Borg score	Weekly
Self-reported questionnaires	FACITF-4^j^	Weekly

^a^IMU: inertial measurement unit.

^b^Wearing at home for 14 consecutive days between visit 2 and visit 4.

^c^Within 2 weeks after each in-clinic visit.

^d^ESS: Epworth Sleepiness Scale.

^e^MDS-UPDRS-II: Movement Disorders Society–sponsored revision of the Unified Parkinson’s Disease Rating Scale (II) motor experiences of the daily living section.

^f^HADS: Hospital Anxiety and Depression Scale.

^g^PDQ-39: 39-item Parkinson’s Disease Questionnaire.

^h^LAPAQ: Longitudinal Aging Study Amsterdam Physical Activity Questionnaire.

^i^CCQ: Clinical Chronic Obstructive Pulmonary Disease Questionnaire.

^j^FACIT-Fatigue: Functional Assessment of Chronic Illness Therapy.

#### In-Clinic Measurements

The in-clinic assessments will consist of 3 different walking tests. The first test is the 6MWT which will be performed twice according to the most recent guidelines over a straight course of 10 meters [45]. The participants will be instructed to walk at a comfortable speed that they can sustain for 6 minutes. The physiotherapist measures the walked meters during the 6MWT. Second, the TUG will be performed and will be executed twice [46]. Participants will sit in a chair, stand up, walk for 3 meters at a comfortable speed, turn, walk back, and sit down in the chair again. The physiotherapist will measure the time it takes to complete this task. The third walking test is the 10-meter walk test (10MWT) [47]. This test will be performed 6 times; 3 times without cognitive tasks (single context 10MWT) and 3 times with cognitive tasks (dual context 10MWT). During all 6 times, participants will walk over a straight 10-meter course at a comfortable speed with alternating contexts. The physiotherapist will measure the time it takes to walk between the 2nd and 8th meters of the walkway. During the dual context 10MWT, participants will perform a small cognitive task during walking, for example counting backwards from 100 with steps of 7.

#### Home Measurements

Participants will wear the Verily Study Watch for 24 hours a day throughout the whole study. The device collects passively recorded data while participants wear it. Additionally, participants will be asked to complete the virtual walk test (VWT) independently at home. The TUG and 6MWT with pre- and post-Borg scores will be performed once a week with the guidance of the graphical user interface on the Verily Study Watch. The Verily Study Watch will provide a notification to the participant on the chosen preset day and time to remind them to perform the VWT. The watch will record the start and end of the 6MWT and TUG from button presses on the watch by the participant. Participants with PD will be asked to perform the walk tests 30 minutes to an hour after medication intake and give a tag on the Verily Study Watch, while participants with COPD can choose a standardized time unrelated to medication intake. The VWT can be postponed to the next day at the same time if desirable.

Participants will choose a course to perform the 6MWT inside their homes where they can walk straight for as many meters as possible. Throughout this study, participants will use the same course every time they perform the VWT. A map will be sent to the Radboudumc research team containing an overview of the course taken. After each visit, participants will complete a set of validated questionnaires at home, including about quality of life, physical activity, fatigue, sleep, anxiety, and depression in a diagnosis-specific questionnaire [48-55]. These questionnaires will be completed digitally within 2 weeks after each visit. The questionnaire about fatigue will be completed every week.

#### Intervention

Between visit 2 and visit 4 participants will participate in high-intensity aerobic exercise training according to current physiotherapy guidelines [34,35]. With this intervention, we aim to increase the physical capacity of our participants and, more specifically, the participants’ walking capacity to measure the sensitivity to detect changes of the device-assisted 6MWT.

The participants will follow a training session twice a week under the supervision of their physiotherapist. These sessions can be individual sessions or group sessions together with other study participants. Alongside these training sessions, participants will be asked to train independently at home once a week by going outside for a walk. The supervised training sessions will consist of a mix between general physical capacity training and localized muscle function training with a focus on the lower extremities. If needed, the session can be completed with components that are important for the participant, for example, balance training. The physiotherapists will keep a log of each supervised session including the so-called FITT factors: frequency, intensity, timing, and type of training.

### Statistical Analyses

The primary aim is to study the measurement properties of the device-assisted 6MWT to assess its ability to obtain physical capacity at home. Our secondary aim is to study these measurement properties of the device-assisted TUG.

The data from the device-assisted 6MWT will be derived from three different situations: (1) during the in-clinic walk tests, (2) during the VWT at home administered via application of the watch, and (3) during free-living where the data are passively recorded.

For our primary aim, we first obtain the measurement agreement between the in-clinic 6-minute walk test distance (6MWD) derived from the Verily Study Watch and the measurements by the physiotherapist. This will be assessed using the intraclass correlation coefficient (ICC) and will be obtained for every study visit. Further, the measurement agreement between the 6MWD of the last virtual 6MWT before the in-clinic assessments and the measurements by the physiotherapist in-clinic will be assessed with the ICC. A Bland-Altman plot will be provided as well. The measurement agreement will be considered “good” when the ICC is at least 0.70 [56].

Second, the test-retest reliability of the 6MWT taken at different visits will be assessed by reporting the ICC. Here, we will use the 6MWD derived from the Verily Study Watch of the 2 in-clinic 6MWTs performed during all 4 visits. The test-retest reliability of the weekly 6MWT at home will also be obtained using the ICC. A Bland-Altman plot will be provided to assess the test-retest reliability too.

Third, we will characterize the performance of the device-assisted 6MWT in capturing changes over time with the physiotherapy intervention. For this comparison, we will use the 6MWD derived from the device-assisted 6MWT during visit 2 and visit 4.

For our secondary aim is the measurement agreement between the in-clinic TUG obtained with the Verily Study Watch and the measurements by the physiotherapist. We will assess this for every study visit using the ICC. Further, the measurement agreement between the last virtual TUG before the in-clinic assessments and the measurements by the physiotherapist will be assessed with the ICC. A Bland-Altman plot will be provided as well.

The TUG derived from the Verily Study Watch of the 2 in-clinic TUGs will be used to obtain the test-retest reliability during all 4 visits. The test-retest reliability of the weekly TUG at home will also be obtained using the ICC. A Bland-Altman plot will be provided too.

Finally, we will characterize the performance of the sensitivity of the device-assisted TUG in capturing changes over time after the physiotherapy intervention. For this comparison, we will use the TUG time derived from the device-assisted TUG during visit 2 and visit 4.

As an exploratory aim, the passively recorded data will be used to relate clinical assessments and free-living–based metrics to patient-reported outcomes.

### Sample Size Consideration

No formal sample size calculation was performed. Prior research states that a sample size of at least 50 participants is recommended for validity studies wherein correlation coefficients are estimated [57]. However, larger sample sizes are preferred especially in populations with chronic conditions. Given the fact that we use the ICC as the end point of our primary aim and potential loss of data due to dropouts or device failure, 100 participants with PD and 100 participants with COPD will provide a reasonable sample size for the characterization of performance.

### Ethical Considerations

This study is being conducted in conformance with the Good Clinical Practice ICH E6 guideline. Further, this study is conducted in compliance with the Ethical principles for medical research involving human subjects as defined in the Declaration of Helsinki (2007-2008), the Dutch Personal Data Protection Act, and the ISO 14155:2020 (Medical devices). The Medisch Ethische Toetsing Comissie Arnhem Nijmegen approved this study’s protocol and communication materials (2021-13165; NL78292.091.21). Written informed consent is obtained before any study procedure is performed. Participants who are not able to give written consent will be excluded from participation.

## Results

In total, 89 people with PD and 65 people with COPD were included in this study. Data analysis will start in April 2024, with the first results of the analysis expected in the fourth quarter of 2024.

## Discussion

The purpose of the ADAPT study is to evaluate the measurement properties of a device-assisted 6MWT and a device-assisted TUG for assessing physical capacity in the daily lives of people with PD and COPD.

Currently, the 6MWT is used to measure physical capacity but it is performed in clinical settings, where the circumstances are always optimized. That makes it hard to obtain a representative view of a person’s physical capacity in their own environment. Wearable technology may offer a solution that enables us to better understand true physical functioning in daily life and its fluctuations throughout the day when deployed at home [23]. Therefore, it is relevant to investigate the measurement properties of a home-based 6MWT when using a wearable device.

This study will lead to unique insights for several reasons. First, the collected data using both regular assessments and continuous data collection using wearable sensors will give us important insights into daily life functioning. Another unique element is the broad inclusion criteria, that allow us to collect data from “real-life” patients and to obtain a more representative view of the true population. Besides that, the repeated measurements in the clinic but also at home and the passively recorded data provide insight into a patient’s functioning in daily life. Those include fluctuations regarding medication intake and several other environmental or personal factors, like heart rate and quality of sleep.

The design of this study is complex, which creates potential challenges. This includes the complex logistic distribution of study materials and this study’s coordination throughout the country. Using 2 different devices that both have different ways regarding preparation and setup before data collection can take place is also challenging, for both participants and physiotherapists. We have addressed this issue by extensively training the physiotherapists and creating videos about the devices for participants. In case of emergencies or questions, we are easily reachable by email and phone for both physiotherapists and participants.

Another challenge will be the inclusion of participants since we only approach patients who are under treatment within the trained physiotherapists’ clinics. Should the inclusion of participants stagnate, we will use our own network to approach participants and see if they are willing to travel to an affiliated practice near their homes. However, even with these solutions, we are aware that participants may feel insecure or not skilled enough about using the devices and decide not to participate or even stop participation. This is compounded by the fact that the vast majority of both people with PD and people with COPD are older.

We also expect a challenge in the analysis of the 6MWT measurements at home. Every participant chooses their own course indoors, which leads to an enormous variety in course shapes, course lengths, and number of turns. Not every participant can walk straight for 10 meters indoors, leading to a shorter course compared to the in-clinic measurements. The study from Fell et al [58] showed that a shorter pathway reduces the 6MWD compared to the traditional 30-meter pathway. We chose to use the 10-meter course to ensure comparability with the 6MWT performed at home. It is more likely that participants can walk for 10 meters straight in their homes than to walk for 30 meters. To gain insight into the courses of our participants, they will create a map of their homes in which they draw their 6MWT course and add the distances of several components of their course.

Additionally, there might be a potential influence of disease severity in the PD cohort when determining the 6MWD from the Verily Study Watch. It is known that people with PD have a significantly reduced step length compared to healthy controls [59]. When analyzing the data, the step length of the participants needs to be defined. The equations used in healthy populations to estimate a person’s step length based on body height and age might not be appropriate here. A sensitivity analysis will be performed during the data analysis to check if participants with higher disease severity (ie, a higher Movement Disorders Society–sponsored revision of the Unified Parkinson’s Disease Rating Scale part III score) will have a worse correlation between the 6MWD from the Verily Study Watch compared to the measurements of the physiotherapists.

This study design also serves as an important feasibility test for remote VWTs. In recent years, the 6MWT and TUG have been studied at home in people with cardiovascular, pulmonary, and neurological diseases [25,60-62]. Different approaches were used to measure the distance walked and time to complete both tests, for example with wearable sensors [19,63], smartphone apps [25,64-66], and more traditional tools like a rope and a stopwatch [67,68]. Based on their results, administering physical capacity tests at home seems to be a promising tool for clinical management or research. However, these tests should be uncomplicated to perform safely and require little space. An example of a home-based test can be the Parkinson’s Disease Virtual Motor Exam which has been deployed to remotely measure the severity of tremor, bradykinesia, and gait impairment [39]. The data from this latter study suggest that people with PD engage with the Parkinson’s Disease Virtual Motor Exam and can complete remote active assessments of motor function and yield data of sufficient quality [15]. Since we use the same smartwatch and user interface but with different tests, we expect that the quality of the data is sufficient for our purposes.

With the results of this study, we will gain insight into the measurement properties of the device-assisted 6MWT and TUG in the clinic and at home. When reliable and valid, we will be able to digitally assess a person’s physical capacity performed in their own familiar environment and capture daily life functioning. This can contribute to better information that makes it possible to personalize treatment options.

## Abbreviations

6MWD: 6-minute walk test distance
6MWT: 6-minute walk test
10MWT: 10-meter walk test
ADAPT: Accuracy of Digital Assessment of Performance Trial
CE: Conformité Européenne
COPD: chronic obstructive pulmonary disease
FITT: frequency, intensity, timing, and type of training
ICC: intraclass correlation coefficient
PD: Parkinson disease
TUG: Timed Up and Go test
VWT: virtual walk test
