# Correction: Measuring Physical Functioning Using Wearable Sensors in Parkinson Disease and Chronic Obstructive Pulmonary Disease (the Accuracy of Digital Assessment of Performance Trial Study): Protocol for a Prospective Observational Study

**DOI:** 10.2196/68647

**Published:** 2024-12-09

**Authors:** Debbie de Graaf, Nienke M de Vries, Tessa van de Zande, Janneke J P Schimmel, Sooyoon Shin, Nathan Kowahl, Poulami Barman, Ritu Kapur, William J Marks Jr, Alex van 't Hul, Bastiaan R Bloem

**Affiliations:** 1 Radboud University Medical Center Donders Institute for Brain, Cognition and Behavior Department of Neurology, Center of Expertise for Parkinson & Movement Disorders Nijmegen Netherlands; 2 Verily Life Sciences South San Fransisco, CA United States; 3 Radboud University Medical Center, Radboud Institute for Health Sciences Department of Respiratory Diseases Nijmegen Netherlands

In “Measuring Physical Functioning Using Wearable Sensors in Parkinson Disease and Chronic Obstructive Pulmonary Disease (the Accuracy of Digital Assessment of Performance Trial Study): Protocol for a Prospective Observational Study” (JMIR Res Protoc 2024 May 7:13:e55452) the authors noticed one error:

The last author's name was listed as:

Bastiaan Bloem

This has been changed to the following:

Bastiaan R Bloem

The correction will appear in the online version of the paper on the JMIR Publications website on December 09, 2024, together with the publication of this correction notice. Because this was made after submission to PubMed, PubMed Central, and other full-text repositories, the corrected article has also been resubmitted to those repositories. 

